# Transient Dynamic Operation of G-Quadruplex-Gated Glucose Oxidase-Loaded ZIF-90 Metal–Organic Framework Nanoparticle Bioreactors

**DOI:** 10.1021/acs.nanolett.3c02542

**Published:** 2023-09-05

**Authors:** Yunlong Qin, Yu Ouyang, Jianbang Wang, Xinghua Chen, Yang Sung Sohn, Itamar Willner

**Affiliations:** † The Institute of Chemistry, The Hebrew University of Jerusalem, Jerusalem 91904, Israel; ‡ The Institute of Life Science, The Hebrew University of Jerusalem, Jerusalem 91904, Israel

**Keywords:** DNA, Chemiluminescence, Dissipative circuit, DNAzyme, Biocatalytic cascade, Strand displacement

## Abstract

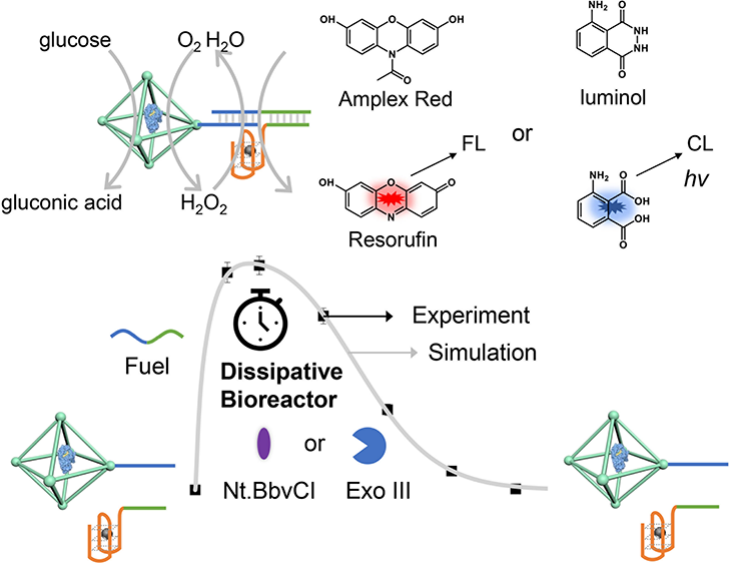

Glucose oxidase-loaded ZIF-90 metal–organic framework nanoparticles conjugated to hemin-G-quadruplexes act as functional bioreactor hybrids operating transient dissipative biocatalytic cascaded transformations consisting of the glucose-driven H_2_O_2_-mediated oxidation of Amplex-Red to resorufin or the glucose-driven generation of chemiluminescence by the H_2_O_2_-mediated oxidation of luminol. One system involves the fueled activation of a reaction module leading to the temporal formation and depletion of the bioreactor conjugate operating the nickase-guided transient biocatalytic cascades. The second system demonstrates the fueled activation of a reaction module yielding a bioreactor conjugate operating the exonuclease III-dictated transient operation of the two biocatalytic cascades. The temporal operations of the bioreactor circuits are accompanied by kinetic models and computational simulations enabling us to predict the dynamic behavior of the systems subjected to different auxiliary conditions.

Emulating native transient dissipative circuits by synthetic networks attracts substantial research efforts within the rapidly developing area of systems chemistry.^1−3^ Particularly the information encoded in the base sequence of nucleic acids provides a versatile means to dynamically reconfigure the biopolymer by a rich “tool-box” of triggers into diverse structural motives.^4^ Indeed, diverse dynamic reversible reconfiguration processes of nucleic acids, such as strand displacement,^5,6^ formation and dissociation of G-quadruplex,^7,8^ stabilization/destabilization of duplex nucleic acids by metal ions,^9−11^ light-stimulated stabilization and separation of nucleic acid duplexes,^12,13^ and enzymatic manipulation of the oligonucleotide biopolymer,^14−19^ were used to develop DNA switches^20,21^ and machines,^22,23^ logic gates,^24^ programmed DNA nanostructures,^25−27^ and switchable DNA-based materials.^28−30^ Moreover, the integration of control units within the switchable reconfigurable DNA circuits provides a means to develop transiently operating dissipative nucleic acid-based networks.^31−33^ Different triggers controlling the temporal transient operation of the circuits were introduced, including the application of enzymes, such as endonuclease,^34^ nickase,^35^ or ligase,^36,37^ as catalytic units regulating the formation and temporal depletion of DNA structures, the application of DNAzymes,^38^ the ion-induced formation and dissociation of G-quadruplexes,^39^ the ribonuclease (RNase)-stimulated control over transcription machineries,^40^ or the application of light-signals as temporal regulating stimuli of DNA circuits.^38,41^ Identification of useful applications of transient dissipative DNA circuits is, however, still challenging. Several applications of transient nucleic acid-based circuits were demonstrated, including the temporal aggregation/deaggregation of metal nanoparticles or semiconductor quantum dots and control over their optical properties of the systems,^42,43^ the temporal formation and separation of DNA nanostructures, such as microtubules or wires,^44^ and the conjugation of the DNA circuits to enzymes, driving transient biocatalytic cascades.^45^

Development of catalytic nanoparticles,^46−48^ such as metal,^49−52^ metal oxide,^53−58^ metal–organic framework nanoparticles,^59−63^ or carbon-based nanoparticles,^64−66^ “nanozymes”, attracts substantial research efforts in catalysis and materials science. Also, integration of biocatalysts and catalytic nanoparticles into nanoparticle frameworks generated hybrid composites acting as bioreactor systems driving catalytic cascades.^67^ Tethering of aptamer strands to nanozymes led to aptananozymes revealing the binding and catalytic functions mimicking native enzymes^68^ and to cell-targeting catalytic nanoparticles for therapeutic applications.^69^ Moreover, catalytic nanoparticles conjugated to catalytic nucleic acids, DNAzymes, acted as hybrids operating as bioreactor systems driving catalytic or biocatalytic cascades.^70^ Diverse applications of nucleic acid-modified nanozymes were reported for sensing,^71−73^ imaging,^74,75^ and therapeutic uses.^69,76−78^ Particularly, the loading of metal–organic framework nanoparticles with nanoparticle catalysts,^79^ biocatalyst,^80^ or drugs^81^ and capping the loaded nanocarriers with stimuli-responsive nucleic acid gates^82^ for controlled release of the loads were demonstrated.

Accordingly, the conjugation of transiently operating nucleic acid gates to nanozymes could introduce dynamic dimensions to nucleic acid/nanozyme hybrids. Here, we report on the assembly of supramolecular bioreactor systems consisting of nucleic acid-modified glucose oxidase (GOx)-loaded ZIF-90 metal–organic framework nanoparticles (NMOFs) and hemin-G-quadruplex constituents demonstrating transient-peroxidase like activities in the presence of the nickase, Nt.BbvCI, or exonuclease III, Exo III, as control units. The ZIF-90 nanoparticles were selected due to the ease to integrate GOx into the ZIF framework and due to the surface carboxaldehyde functionalities of ZIF-90 that allow the covalent conjugation of the nucleic acids to the scaffold.^70^

## Transient Nickase-Driven Biocatalytic Cascades Using GOx-Loaded/Hemin-G-Quadruplex-Conjugated ZIF-90 as Functional Frameworks

**Figure 1:**
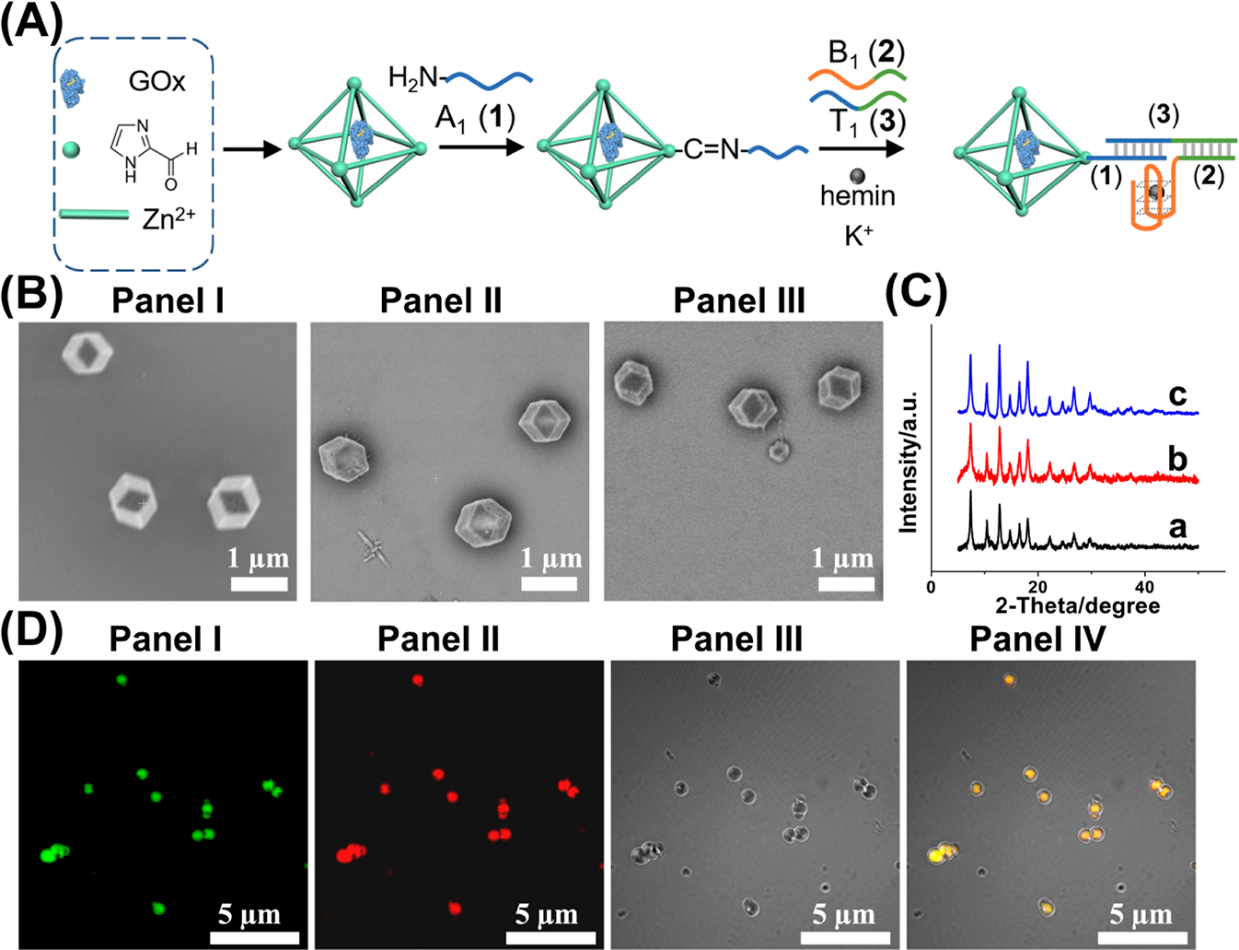
(A) Schematic synthesis of the (**1**)+(**2**)/(**3**) supramolecular GOx-loaded ZIF-90 NMOFs/hemin-G-Quadruplex bioreactor particles. (B) SEM images corresponding to panel I, bare ZIF-90 NMOFs; panel II, GOx-loaded ZIF-90 NMOFs; panel III, (**1**)+(**2**)/(**3**) supramolecular GOx-loaded ZIF-90 NMOFs. (C) XRD spectra corresponding to (a) bare ZIF-90 NMOFs; (b) GOx-loaded ZIF-90 NMOFs; (c) (**1**)+(**2**)/(**3**) modified GOx-loaded ZIF-90 NMOFs. (D) Confocal microscopy images corresponding to (**1**)+(**2**)/(**3**) supramolecular nanoparticles consisting of FITC-modified-GOx-loaded NMOFs and Zn(II)-protoporphyrin IX (Zn(II)PPIX)-modified G-quadruplex units: panel I, fluorescence confocal microscopy image of the NMOFs using the FITC fluorescence channel, λ = 420 nm; panel II, fluorescence confocal microscopy image of the NMOFs using the Zn(II)PPIX/G-quadruplex fluorescence channel, λ = 590 nm; panel III, bright-field microscopy image; panel IV, overlay of panels I–III.

Figure 1 depicts the synthesis and characterization of the (**1**)+(**2**)/(**3**) supramolecular GOx-loaded ZIF-90 NMOFs hybrid as the functional conjugate for the nickase-activated, transient bioreactor system. Glucose oxidase (GOx)-loaded ZIF-90 NMOFs were prepared according to the literature^70^ by reacting imidazole-2-carboxaldehyde with Zn^2+^-ion, in the presence of GOx, Figure 1A. The carboxaldehyde functions associated with the NMOFs were functionalized with amino-nucleic acid tethers A_1_, (**1**), that provide the anchoring sites for the assembly of the GOx-loaded ZIF-90 NMOFs/G-quadruplex conjugate. Reacting the (**1**)-functionalized NMOFs with the strand T_1_, (**3**), and the G-rich strand B_1_, (**2**), in the presence of K^+^-ion yielded the GOx-loaded ZIF-90 NMOFs/G-quadruplex hybrid, and the subsequent treatment of the assembly with hemin yielded the GOx-loaded ZIF-90 NMOFs/hemin-G-quadruplex bioreactor hybrid system. Figure 1B depicts the SEM images of bare ZIF-90 NMOFs, the GOx-loaded ZIF-90 NMOFs, and the (**1**)+(**2**)/(**3**)-modified GOx-loaded ZIF-90 NMOFs. Identical dodecahedral crystalline NMOFs are observed, indicating that the functionalization of the NMOFs with the biomaterials does not affect the crystalline structure of the particles. This is further supported by identical XRD patterns of the NMOFs in the different states of modification of the NMOFs (Figure 1C). The loading of the NMOFs with GOx corresponded to 82 μg/mg NMOFs, and the loading of the tether (**1**) on the NMOFs and of the G-quadruplexes on the NMOFs both corresponded to 10 nmol/mg (for experimental details evaluating the loading of the components on the NMOFs see Supporting Information, Figures S1–S3). Confocal microscopy imaging experiments, Figure 1D, further supported the construction of the (**1**)+(**2**)/(**3**) supramolecular GOx-loaded ZIF-90 NMOFs/G-quadruplex conjugate. The loading of the hybrid with FITC (fluorescein isothiocyanate isomer I)-modified GOx demonstrated green fluorescent (λ = 420 nm) particles, panel I, consistent with the GOx-loaded NMOFs. Subjecting the particles to Zn(II) protoporphyrin IX, Zn(II)PPIX, resulted in the characteristic red fluorescence, λ = 590 nm, of Zn(II)PPIX bound to G-quadruplexes,^83^ panel II. The bright-field image of the particles is depicted in panel III, and the overlay of the images is displayed in panel IV, revealing a yellow fluorescence of the combined constituents associated with the hybrid NMOFs. The successful construction of (**1**)+(**2**)/(**3**) supramolecular GOx-loaded ZIF-90 NMOFs/hemin-G-quadruplex bioreactor was further characterized by the efficient biocatalytic cascades of glucose-driven H_2_O_2_-channeled oxidation of Amplex-Red or generation of chemiluminescence, Figure S4. (We note that the added K^+^-ion is essential to assemble the (**1**)+(**2**)/(**3**) supramolecular GOx-loaded ZIF-90 NMOFs/hemin-G-quadruplex bioreactor; see Figure S4B, panels I and II, curve c.)

**Figure 2:**
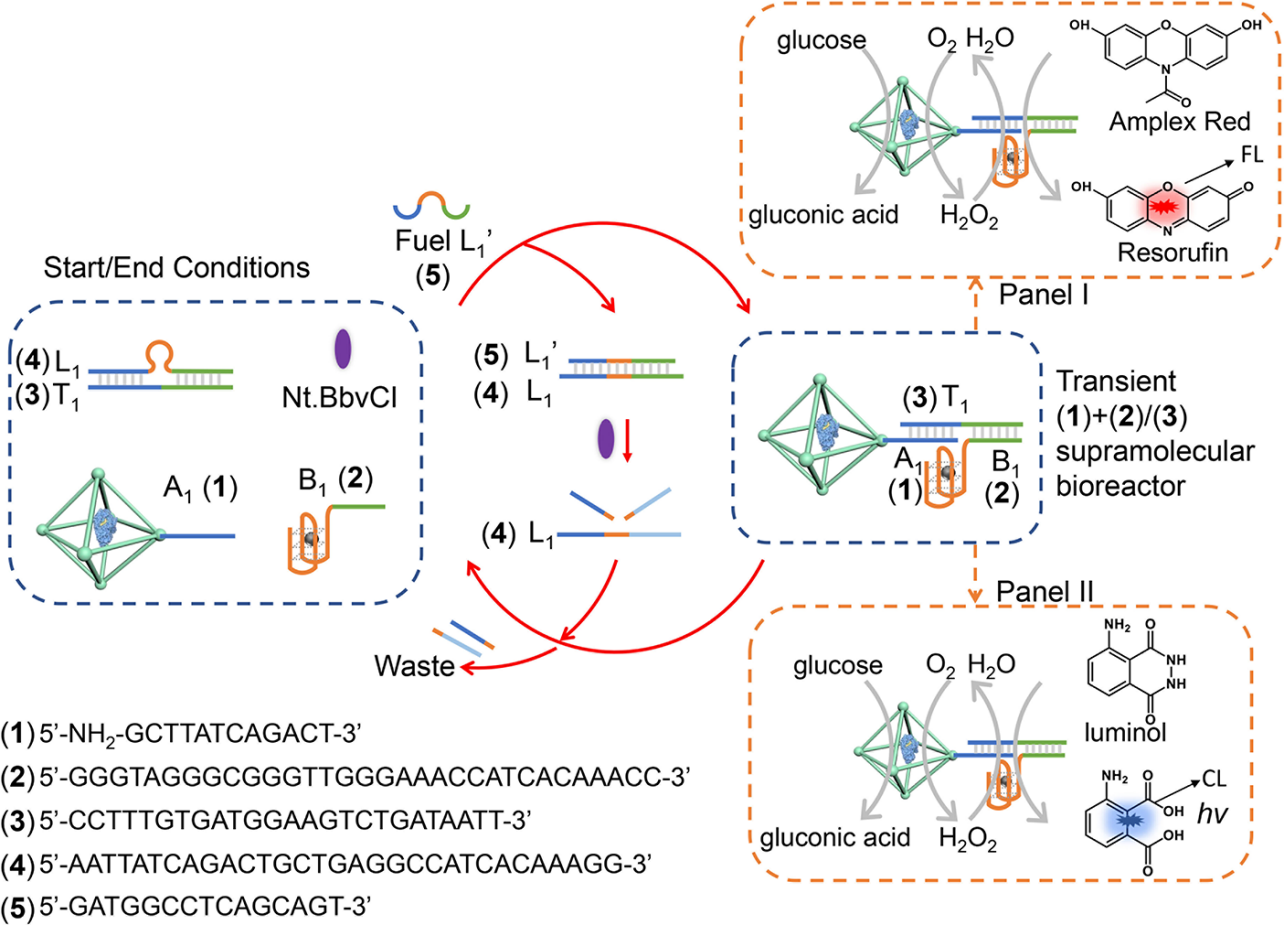
Schematic reaction module for the fueled transient operation of the (**1**)+(**2**)/(**3**) GOx-loaded ZIF-90 NMOFs/hemin-G-quadruplex bioreactor system leading to the temporal biocatalytic cascades consisting of the following: panel I, GOx/hemin-G-quadruplex catalyzed oxidation of Amplex-Red to fluorescent resorufin; panel II, GOx/hemin-G-quadruplex catalyzed generation of chemiluminescence through the catalyzed H_2_O_2_ oxidation of luminol.

Figure 2 depicts schematically the fuel-driven nickase-modulated transient operation of the (**1**)+(**2**)/(**3**) GOx-loaded ZIF-90 NMOFs/hemin-G-quadruplex bioreactor system. The initial reaction module consists of the A_1_, (**1**)-functionalized GOx-loaded ZIF-90 NMOFs, the hemin-G-quadruplex, B_1_, (**2**) constituent, the duplex composed of L_1_/T_1_, (**4**)/(**3**), and the nicking enzyme Nt.BbvCI. Subjecting the reaction module to the fuel strand L_1_′, (**5**) results in the displacement of the duplex L_1_/T_1_, (**4**)/(**3**) to yield the duplex L_1_/L_1_′, (**4**)/(**5**) and free T_1_, (**3**). The released T_1_, (**3**) self-assembles the (**1**)+(**2**)/(**3**) GOx-loaded ZIF-90 NMOFs/hemin-G-quadruplex bioreactor hybrid system that drives two different biocatalytic cascades. One biocatalytic cascade, panel I, involves the aerobic oxidation of glucose, yielding gluconic acid and H_2_O_2_ and the subsequent hemin-G-quadruplex-catalyzed oxidation of Amplex-Red by H_2_O_2_ to form the fluorescent resorufin. The second biocatalytic cascade, driven by the GOx-loaded ZIF-90 NMOFs/hemin-G-quadruplex bioreactor, is displayed in Figure 2, panel II and involves the aerobic oxidation of glucose to gluconic acid and H_2_O_2_ and the subsequent hemin-G-quadruplex-catalyzed oxidation of luminol by the H_2_O_2_ to generate chemiluminescence, λ = 425 nm.^84^ The resulting duplex L_1_/L_1_′, (**4**)/(**5**), generated upon the fueled operation of the reaction module and the formation of the hybrid NMOFs bioreactor, is engineered to include in the sequence of L_1_′, (**5**) the nicking site to be cleaved by Nt.BbvCI. Cleavage of the L_1_′, (**5**) leads to fragmented “waste” products that are separated from the duplex, leading to the separation of L_1_, (**4**). The separated strand L_1_, (**4**) displaces T_1_, (**3**) from the (**1**)+(**2**)/(**3**) hybrid bioreactor structure, resulting in the separation of two bioreactor constituents, the blockage of the two biocatalytic cascades displayed in panels I and II, and the regeneration of the parent inactive reaction module. That is, the L_1_′, (**5**) triggered activation of the reaction module leads to the temporal activation of the two biocatalytic cascades that reveal a guided transient operation and depletion mechanism to regenerate the parent inactive state.

**Figure 3:**
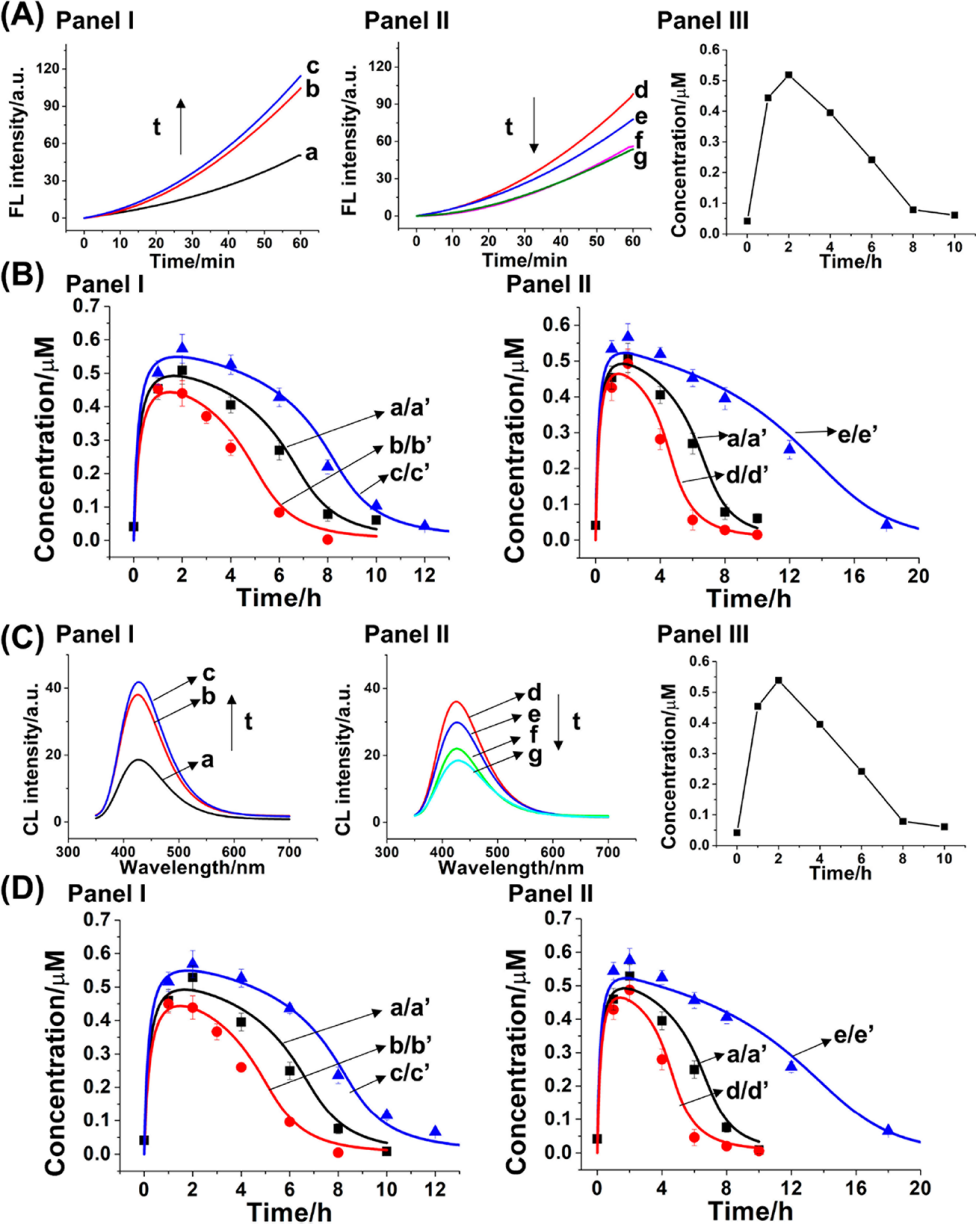
(A) Time-dependent fluorescence changes of resorufin generated by samples withdrawn, at time intervals, from the dynamic reaction module depicted in panel I of Figure 2: panel I, samples withdrawn at (a) *t* = 0 h, (b) *t* = 1 h, (c) *t* = 2 h; panel II, samples withdrawn at (d) *t* = 4 h, (e) *t* = 6 h, (f) *t* = 8 h, (g) *t* = 10 h; panel III, temporal, transient concentration changes of the (**1**)+(**2**)/(**3**) supramolecular structure generating the fluorescent resorufin, upon operation of the dynamic reaction module shown in Figure 2, panel I. The experimental conditions operating the dynamic process shown in panels I–III are A_1_-(**1**), 1 μM; B_1_-(**2**), 1 μM; T_1_/L_1_-(**3**)/(**4**), 2 μM; L_1_′-(**5**), 3 μM; Nt.BbvCI, 0.046 μM. (B) Probing the effects of the concentrations of the fuel strand L_1_′ (panel I) and of the Nt.BbvCI (panel II) on the temporal transient concentrations of the (**1**)+(**2**)/(**3**) supramolecular structure, generating fluorescent resorufin according to Figure 2, panel I. Part B, panel I: (a) Dotted points correspond to experimental data recorded at the conditions specified in (A); (a′) solid curves correspond to computationally simulated results using the kinetic model formulated in Figure S8, Supporting Information. (b′, c′) Computationally simulated transient concentrations of (**1**)+(**2**)/(**3**) supramolecular structure, generating fluorescent resorufin, at auxiliary conditions L_1_′ = 2 and 4 μM, respectively. (b, c) Dotted data correspond to experimentally validated results in the presence of L_1_′ = 2 and 4 μM, respectively. Part B, panel II: (a/a′) is the same condition with (a/a′) in panel I. (d′, e′) Computationally simulated transient concentrations of (**1**)+(**2**)/(**3**) supramolecular structure, generating fluorescent resorufin, at auxiliary conditions Nt.BbvCI = 0.069 and 0.023 μM, respectively. (d, e) Dotted data correspond to experimentally validated results in the presence of Nt.BbvCI = 0.069 and 0.023 μM, respectively. (C) Time-dependent chemiluminescence changes of luminol generated by samples withdrawn at time intervals from the dynamic reaction module depicted in panel II, Figure 2: panel I, samples withdrawn at (a) *t* = 0 h, (b) *t* = 1 h, (c) *t* = 2 h; panel II, samples withdrawn at (d) *t* = 4 h, (e) *t* = 6 h, (f) *t* = 8 h, (g) *t* = 10 h; panel III, temporal, transient concentration changes of the (**1**)+(**2**)/(**3**) supramolecular structure generating chemiluminescence upon operation of the dynamic reaction module shown in Figure 2, panel II. Experimental conditions operating the dynamic process shown in panels I–III are A_1_-(**1**), 1 μM; B_1_-(**2**), 1 μM; T_1_/L_1_-(**3**)/(**4**), 2 μM; L_1_′-(**5**), 3 μM; Nt.BbvCI, 0.046 μM. (D) Probing the effects of the concentrations of the fuel strand L_1_′ (panel I) and the concentrations of the Nt.BbvCI (panel II) on the temporal transient generation of the (**1**)+(**2**)/(**3**) supramolecular structure and generation of chemiluminescence according to Figure 2, panel II. Part D, panel I: (a′) Dotted points correspond to experimental data recorded at the conditions specified in (C); (a′) solid curves correspond to computationally simulated result using the kinetic model formulated in Figure S8, Supporting Information. (b′, c′) Computationally simulated transient concentrations of (**1**)+(**2**)/(**3**) supramolecular structure generating chemiluminescence at auxiliary conditions L_1_′ = 2 and 4 μM, respectively. (b, c) Dotted data correspond to experimentally validated results in the presence of L_1_′ = 2 and 4 μM, respectively. Part D, panel II: (a/a′) is the same condition with (a/a′) in panel I. (d′, e′) Computationally simulated transient concentrations of (**1**)+(**2**)/(**3**) supramolecular structure generating chemiluminescence at auxiliary conditions Nt.BbvCI = 0.069 and 0.023 μM, respectively. (d, e) Dotted data correspond to experimentally validated results in the presence of Nt.BbvCI = 0.069 and 0.023 μM, respectively.

Panels I and II of Figure 3A display the time-dependent fluorescence changes of resorufin generated at time intervals of the L_1_′-triggered operation of the reaction module (L_1_′ = 3 μM and Nt.BbvCI = 0.046 μM). The rates of resorufin formation increase for a time interval of 2 h and then decrease for a depletion time interval for 10 h leading to regeneration of the parent system. Using a calibration curve relating the fluorescence intensities of resorufin to the concentrations of (**1**)+(**2**)/(**3**) supramolecular structure (Figure S6, Supporting Information), the transient concentrations of (**1**)+(**2**)/(**3**) supramolecular bioreactor, corresponding to the transient catalytic formation of resorufin, were calculated, and these are displayed in Figure 3A, panel III. A transient behavior of the catalytic rates generating resorufin is, indeed, observed. The transient supramolecular catalytic bioreactor generating resorufin is anticipated to be controlled by the concentration of the fuel L_1_′ triggering the reaction module and by the concentration of the nicking enzyme Nt.BbvCI. Qualitatively, increasing the concentration of the fuel strand L_1_′ is anticipated to enhance and enrich the content of the transiently formed resorufin whereas elevating the content of the Nt.BbvCI is expected to decrease the peak content of the catalytically generated resorufin and to shorten the depletion time-interval of the temporal process. The efficiency of the GOx-loaded ZIF-90/hemin-G-quadruplex biocatalytic cascade is controlled by the loading of GOx in the NMOFs and the resulting H_2_O_2_ generated by the GOx-catalyzed aerobic oxidation of glucose. The results are displayed in Figure S5 and accompanied discussion. As the loading of GOx increases, the biocatalytic cascade is enhanced, yet the background signal of the separated GOx-loaded ZIF-90/hemin-G-quadruplex constituents is intensified.

As an attempt to understand the effect of these auxiliary parameters on the transient process, we formulated a kinetic model that follows the transient process, Figure S8. This model was adopted to simulate the experimental results (dotted transient points a) in Figure 3B, panel I, with a computationally fitted transient (solid transient curve a′), generated by the set of rate constants comprising the model, that are summarized in Table S1 (for a detailed description of the simulation process see Figure S8). This set of rate-constants has a value provided that it can predict the transient behavior of the supramolecular bioreactor at other auxiliary conditions. Accordingly, the transient behavior of the formation and depletion of (**1**)+(**2**)/(**3**) supramolecular bioreactor generating fluorescent resorufin was predicted at different auxiliary concentrations of L_1_′ (curves b′ and c′, Figure 3B, panel I) and the nicking enzyme concentrations (curves d′ and e′, Figure 3B, panel II). The predicted results were experimentally validated, curves b and c in Figure 3B, panel I and curves d and e in Figure 3B, panel II, respectively. Very good fit between the experimental results and the computationally predicted transients is demonstrated, supporting the kinetic model.

Similarly, Figure 3C, panels I and II, depicts the temporal chemiluminescence spectra generated by samples withdrawn at time intervals from the transient operating bioreactor system according to Figure 2, panel II. The L_1_′-triggered activation of the reaction module yields in the first 2 h of operating the bioreactor system reaction samples where the resulting chemiluminescence is temporally intensified, and afterward the bioreactor samples withdrawn from the reaction system show a continuous temporal decrease in the chemiluminescence, reaching the parent chemiluminescence intensities after a time interval of ∼10 h. These results are consistent with the temporal L_1_′-triggered buildup of the (**1**)+(**2**)/(**3**) supramolecular GOx-loaded NMOFs/hemin-G-quadruplex catalytic bioreactor system that undergoes transient depletion by the nickase-induced separation of the bioreactor conjugate, leading to the recovery of the original reaction module. The transient concentration changes of (**1**)+(**2**)/(**3**) supramolecular bioreactor generating chemiluminescence are displayed in Figure 3C, panel III. As before, decreasing the concentration of the fuel strand L_1_′ that activates the reaction module results in lower temporally generated yields of the bioreactor conjugate, leading to lower chemiluminescence intensities, Figure 3D, panel I. Also, increasing the concentration of the nicking enzyme results in lower yields of chemiluminescence generated by the bioreactor system and enhanced depletion rates of the transiently operated bioreactor intermediate, Figure 3D, panel II.

## Transient Exonuclease III-Driven Biocatalytic Cascades Using GOx-Loaded/Hemin-G-Quadruplex-Conjugated ZIF-90 as Functional Frameworks

**Figure 4:**
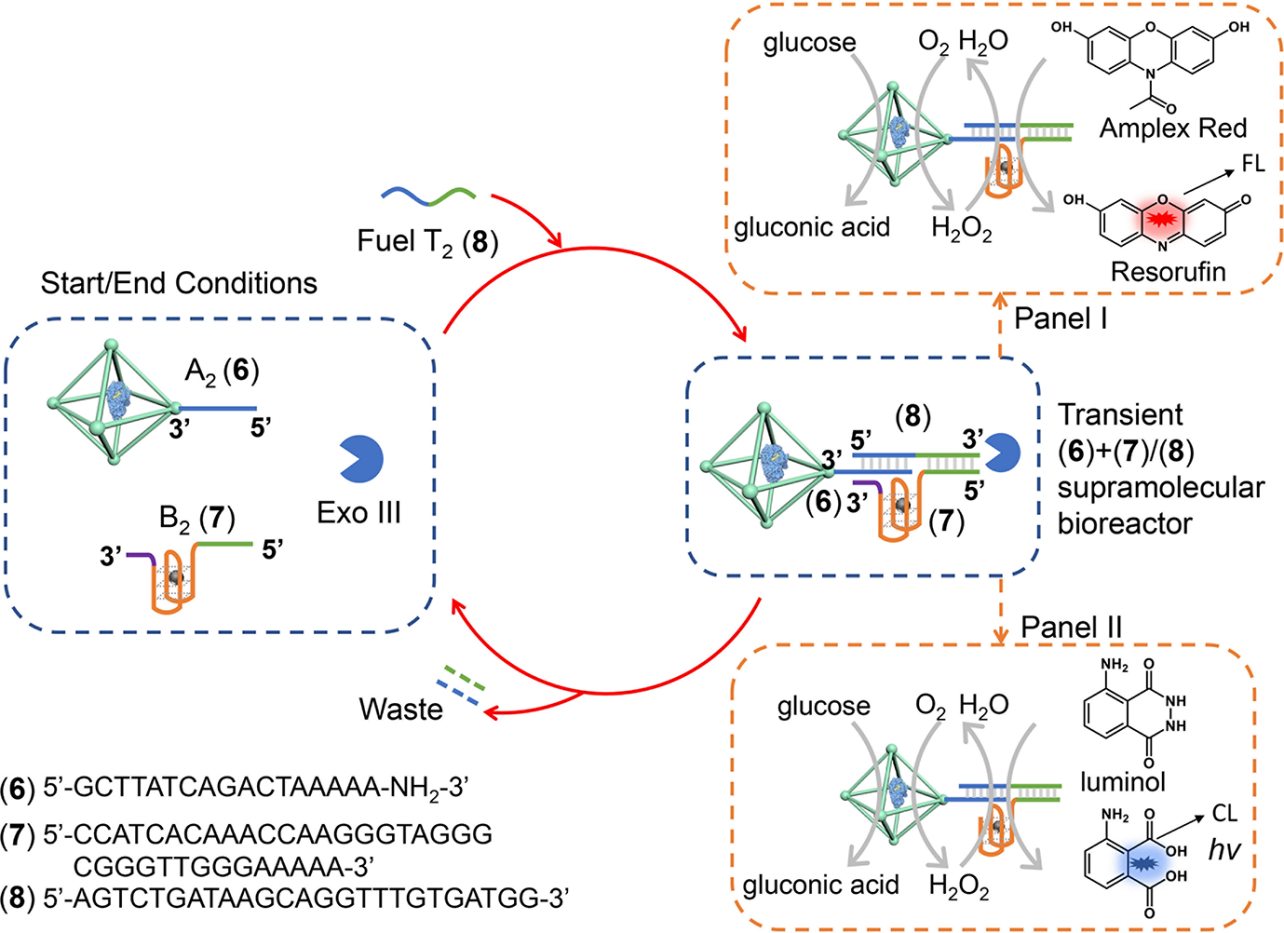
Schematic reaction module for the fueled transient operation of the Exo III-guided (**6**)+(**7**)/(**8**) GOx-loaded ZIF-90 NMOFs/hemin-G-quadruplex bioreactor system leading to the temporal biocatalytic cascades consisting of the following: panel I, GOx/hemin-G-quadruplex catalyzed oxidation of Amplex-Red to fluorescent resorufin; panel II, GOx/hemin-G-quadruplex catalyzed generation of chemiluminescence through the catalyzed H_2_O_2_ oxidation of luminol.

An alternative transient GOx-loaded ZIF-90 NMOFs/hemin-G-quadruplex reaction module leading to the fuel-triggered and transient operation of the bioreactor catalyzing the biocatalytic oxidation of Amplex-Red to resorufin or the biocatalytic generation of chemiluminescence is depicted in Figure 4. In this system, exonuclease III, Exo III, acts as the enzyme controlling the dissipative, transient operation of the bioreactor. The reaction module consists of the GOx-loaded ZIF-90 NMOFs functionalized with the nucleic acid A_2_, (**6**) (loading of GOx 82 μg/mg NMOFs; loading of A_2_ 10 nmol/mg NMOFs), and the hemin-G-quadruplex B_2_, (**7**). The enzyme Exo III is also included in the reaction module. Subjecting the reaction module to the fuel strand T_2_, (**8**), results in the assembly of the (**6**)+(**7**)/(**8**) supramolecular bioreactor complex consisting of the (**8**)-bridged GOx-loaded ZIF-90 NMOFs and hemin-G-quadruplex constituents. The supramolecular complex operates two different biocatalytic cascades. One biocatalytic cascade, panel I, involves the aerobic GOx-catalyzed oxidation of glucose to yield gluconic acid and H_2_O_2_ and the subsequent hemin-G-quadruplex-catalyzed oxidation of Amplex-Red to resorufin. The second biocatalytic cascade driven by the (**6**)+(**7**)/(**8**) supramolecular GOx-loaded ZIF-90 NMOFs/hemin-G-quadruplex bioreactor involves the aerobic GOx-loaded ZIF-90 NMOFs-catalyzed oxidation of glucose to gluconic acid and H_2_O_2_ and the subsequent hemin-G-quadruplex catalyzed oxidation of luminol by H_2_O_2_ and the generation of chemiluminescence, panel II. The Exo III included in the system selectively degrades the 3′-ended fuel strand T_2_, leading to the separation of the supramolecular complex regenerating the rest of the reaction module and producing base fragments T_2_ as waste products. The separation of the supramolecular complex by Exo III leads to the temporal activation of the biocatalytic cascades shown in panels I and II and to the recovery of the parent reaction module. That is, the T_2_-triggered activation of the reaction module leads to the temporal and transient formation of the (**6**)+(**7**)/(**8**) supramolecular complex consisting of the GOx-loaded ZIF-90 NMOFs/hemin-G-quadruplex that guides the temporal biocatalytic cascades shown in Figure 4, panels I and II.

It should be noted that in order to operate the Exo III driven transient generation of the bioreactor, the supramolecular GOx-loaded ZIF-90 NMOFs/hemin-G-quadruplex complex had to be engineered to retain an intact, Exo III-resistant structure, to the extent that only the T_2_ that forms the duplex with B_2_ is being digested by Exo III. We find that it is essential to tether to the G-quadruplex B_2_ a single strand 3′-terminated tether; otherwise, the G-quadruplex is also degraded by Exo III.

**Figure 5:**
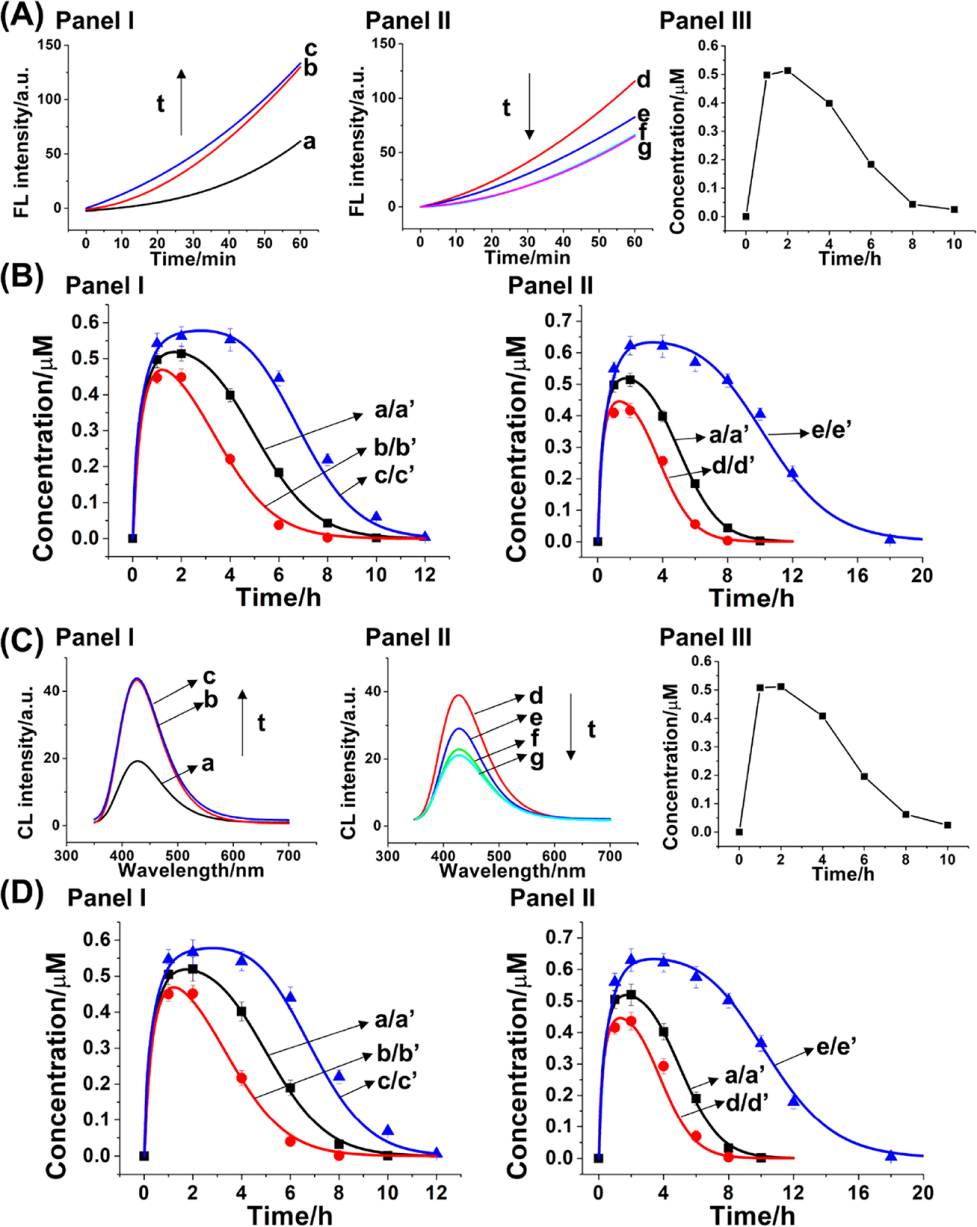
(A) Time-dependent fluorescence changes of resorufin generated by samples withdrawn, at time intervals, from the dynamic reaction module depicted in panel I of Figure 4: panel I, samples withdrawn at (a) *t* = 0 h, (b) *t* = 1 h, (c) *t* = 2 h; panel II, samples withdrawn at (d) *t* = 4 h, (e) *t* = 6 h, (f) *t* = 8 h, (g) *t* = 10 h; panel III, temporal, transient concentration changes of the (**6**)+(**7**)/(**8**) supramolecular structure generating the fluorescent resorufin, upon operation of the dynamic reaction module shown in Figure 4, panel I. The experimental conditions operating the dynamic process shown in panels I–III are A_2_-(**6**), 1 μM; B_2_-(**7**), 1 μM; T_2_-(**8**), 3 μM; and Exo III, 0.4 μM. (B) Probing the effects of the concentrations of the fuel strand T_2_ (panel I) and of the Exo III (panel II) on the temporal transient generation of the (**6**)+(**7**)/(**8**) supramolecular structure, generating fluorescent resorufin according to Figure 4, panel I. Part B, panel I: (a) Dotted points correspond to experimental data recorded at the conditions specified in (A); (a′) solid curves correspond to computationally simulated results using the kinetic model formulated in Figure S11, Supporting Information. (b′, c′) Computationally simulated transient concentrations of (**6**)+(**7**)/(**8**) supramolecular structure, generating fluorescent resorufin, at auxiliary conditions T_2_ = 2 and 4 μM, respectively; (b) and (c) Dotted data correspond to experimentally validated results in the presence of T_2_ = 2 and 4 μM, respectively. Part B, panel II: (a/a′) is the same condition with (a/a′) in panel I. (d′, e′) Computationally simulated transient concentrations of (**6**)+(**7**)/(**8**) supramolecular structure, generating fluorescent resorufin, at auxiliary conditions Exo III = 0.6 and 0.2 μM, respectively. (d, e) Dotted data correspond to experimentally validated results in the presence of Exo III = 0.6 and 0.2 μM, respectively. (C) Time-dependent chemiluminescence changes of luminol generated by samples withdrawn at time intervals from the dynamic reaction module depicted in panel II of Figure 4: panel I, samples withdrawn at (a) *t* = 0 h, (b) *t* = 1 h, (c) *t* = 2 h; panel II, samples withdrawn at (d) *t* = 4 h, (e) *t* = 6 h, (f) *t* = 8 h, (g) *t* = 10 h; panel III, temporal, transient concentration changes of the (**6**)+(**7**)/(**8**) supramolecular structure generating chemiluminescence upon operation of the dynamic reaction module shown in Figure 4, panel II. Experimental conditions operating the dynamic process shown in panels I–III are A_2_-(**6**), 1 μM; B_2_-(**7**), 1 μM; T_2_-(**8**), 3 μM; Exo III, 0.4 μM. (D) Probing the effects of the concentrations of the fuel strand T_2_ (panel I) and the concentrations of the Exo III (panel II) on the temporal transient generation of the (**6**)+(**7**)/(**8**) supramolecular structure and generation of chemiluminescence according to Figure 4, panel II. Part D, panel I: (a) Dotted points correspond to experimental data recorded at the conditions specified in (C); (a′) solid curves correspond to computationally simulated result using the kinetic model formulated in Figure S11, Supporting Information. (b′, c′) Computationally simulated transient concentrations of (**6**)+(**7**)/(**8**) supramolecular structure generating chemiluminescence at auxiliary conditions T_2_ = 2 and 4 μM, respectively. (b, c) Dotted data correspond to experimentally validated results in the presence of T_2_ = 2 and 4 μM, respectively. Part D, panel II: (a/a′) is the same condition with (a/a′) in panel I. (d′, e′) Computationally simulated transient concentrations of (**6**)+(**7**)/(**8**) supramolecular structure generating chemiluminescence at auxiliary conditions Exo III = 0.6 and 0.2 μM, respectively. (d, e) Dotted data correspond to experimentally validated results in the presence of Exo III = 0.6 and 0.2 μM, respectively.

Panels I and II of Figure 5A depict the time-dependent fluorescence changes of resorufin generated upon subjecting the reaction module shown in Figure 4 to the fuel strand T_2_ and withdrawing at time interval samples driving the glucose-initiated H_2_O_2_-mediated oxidation of Amplex Red to resorufin. The withdrawn samples reveal a temporal initial increase in the oxidation rates of Amplex-Red for a time interval of ∼2 h, and afterward, the withdrawn sample shows a continuous temporal decline in the bioreactor catalyzed oxidation rates, which are fully blocked after 8 h, reaching the parent background rates of Amplex-Red oxidation to resorufin by the parent reaction module. Readdition of the fuel strand T_2_ to the system reactivated the transient operation of the reaction module, following the oxidation of Amplex-Red to form resorufin. These results are consistent with the T_2_-fueled/Exo III-guided operation of the reaction module shown in Figure 4. The bioreactor Exo III-driven, T_2_-triggered, transient formation of (**6**)+(**7**)/(**8**) supramolecular bioreactor generating fluorescent resorufin is controlled by the concentrations of T_2_, Figure 5B, panel I, and Exo III, Figure 5B, panel II. As the concentrations of T_2_ increases, the peak rates of temporal resorufin formation are higher, and as the concentration of Exo III increases, the peak rates of catalyzed formation of resorufin are lower and the depletion of the catalytic rates generating resorufin is enhanced. Figure S11 formulates the kinetic model corresponding to the transient operation of the T_2_/Exo III driven (**6**)+(**7**)/(**8**) supramolecular GOx-loaded NMOFs/hemin-G-quadruplex bioreactor system. Figure 5B, panel I, curve a′, depicts the fitted, computationally simulated, temporal concentrations of the catalytic (**6**)+(**7**)/(**8**) supramolecular structure generating resorufin by the T_2_/Exo III-driven bioreactor system, overlapping the experiment transient curve a, using the kinetic models. The derived rate constants following the kinetic model are summarized in Table S2. Using this set of rate constants, the predicted curves corresponding to temporal transient concentrations of the catalytic bioreactor system at different concentrations of T_2_ and Exo III were computed, curves b′ and c′, Figure 5B, panel I, and d′ and e′, Figure 5B, panel II, and the computational results are experimentally validated, curves b and c, Figure 5B, panel I, and curves d and e, Figure 5B, panel II. Indeed, the experimental results fit well to the predicted transient systems. Moreover, the T_2_/Exo III-driven activation of the GOx-loaded ZIF-90 NMOFs/hemin-G-quadruplex bioreactor, Figure 4, panel II, was applied to stimulate the temporally catalyzed generation of chemiluminescence. Panels I and II of Figure 5C depict the temporal chemiluminescence spectra generated by samples withdrawn at time intervals from the T_2_/Exo III-activated reaction module. The triggered reaction module reveals intensified chemiluminescence spectra for a time interval of 2 h and, subsequently, temporal depletion and blockage of the chemiluminescence after 8 h. Figure 5C, panel III shows the temporal, transient concentrations of (**6**)+(**7**)/(**8**) supramolecular structure generating chemiluminescence upon the T_2_/Exo III-triggered activation of the bioreactor system in the presence of 3 μM T_2_ and 0.4 μM Exo III. Figure 5D depicts the experimental and simulated temporal, transient formation of the (**6**)+(**7**)/(**8**) supramolecular bioreactor leading to chemiluminescence upon activation of the reaction module at different concentrations of T_2_ and Exo III. As the concentration of T_2_ increases, the temporal peak chemiluminescence intensity is higher, and as the concentration of Exo III is elevated, the peak chemiluminescence intensity is lower and the dissipative depletion of the bioreactor driven process is enhanced.

## Conclusion

The study introduced nucleic acid-modified/GOx-loaded ZIF-90 conjugates as functional assemblies guiding transient biocatalytic cascades. Such frameworks could find important nanomedical applications for dose-controlled, temporal release of therapeutic agents. For example, the GOx-stimulated activation of the hemin-G-quadruplex peroxidase-mimicking DNAzyme yields reactive oxygen species (ROS) that might act for temporal chemodynamic treatment of cancer cells.^69^ Alternatively, the triggered temporal unlocking of the NMOFs could be used for the dose-controlled release of drugs, e.g., insulin from the NMOFs carriers.^85^ Moreover, the present study introduced the fueled strand displacement principle and coupled enzyme driven transformation as control motives of the dynamic process. Other triggering stimuli for temporal operation of the reaction modules such as miRNA/RNase,^86^ redox-triggered aptamer-ligand complexes,^87^ or light,^38^ may be envisaged.

## Supporting Information Available

Experimental section; calibration curves for determination of the loading of GOx and DNA strands A_1_ or A_2_ involved in the bioreactor; efficient biocatalytic cascades performed by (**1**)+(**2**)/(**3**) supramolecular structure; effect of loading amount of GOx on the transient biocatalytic cascades; calibration curve for determination of the concentration of (**1**)+(**2**)/(**3**) supramolecular structure; kinetic model and simulation details of Nt.BbvCI-guided dissipative bioreactor system; calibration curve for determination of the concentration of (**6**)+(**7**)/(**8**) supramolecular structure; kinetic model and simulation details of Exo III-guided dissipative bioreactor system (PDF)
