# Supplementary material for: Transient Dynamic Operation of G-Quadruplex-Gated Glucose Oxidase-Loaded ZIF-90 Metal–Organic Framework Nanoparticle Bioreactors
Source: Nano Lett. 2023 Sep 5;23(18):8664–73. doi: 10.1021/acs.nanolett.3c02542 (PMC10540265; doi:10.1021/acs.nanolett.3c02542)
Supplement: Supplementary file 1 — nl3c02542_si_001.pdf [file nl3c02542_si_001.pdf]

Supporting information

Transient Dynamic Operation of G-Quadruplex-Gated Glucose Oxidase-loaded ZIF-90 Metal-Organic Framework Nanoparticle Bioreactors

Yunlong Qin,[a] *Yu Ouyang,*[a] *Jianbang Wang,*[a] *Xinghua Chen,*[a] *Yang Sung Sohn,*[b]

Itamar Willner[a*]

[a]The Institute of Chemistry, The Hebrew University of Jerusalem, Jerusalem 91904, Israel. Email: itamar.willner@mail.huji.ac.il.

[b]The Institute of Life Science, The Hebrew University of Jerusalem, Jerusalem 91904, Israel.

Experimental Section

Materials

Imidazole-2-carboxaldehyde (2-ICA) was obtained from Alfa Aesar. Zinc nitrate hexahydrate ($Zn(NO_3)_2 \cdot 6H_2O$), polyvinylpyrrolidone (PVP, MW: 40000), ethanol, glucose oxidase, hemin, glucose, Amplex-Red, luminol, fluorescein isothiocyanate isomer I (FITC) were purchased from Sigma-Aldrich. Nt.BbvCI (10000 units/mL, 2.3 μM) and Exonuclease III (Exo III) (100000 units/mL, 40μM), rCutsmart Buffer (10x) was bought from NEB. GelRed (10000x) was obtained from Biotium. Ultrapure water was obtained by a NANOpure Diamond machine. The DNA strands were ordered from Integrated DNA Technologies and listed below.

(1) A_1: 5’-NH_2-GCTTATCAGACT-3’

(2) B_1: 5’-GGGTAGGGCGGGTTGGGAAACCATCACAAACC-3’

(3) T_1: 5’-CCTTTGTGATGGAAGTCTGATAATT-3’

(4) L_1: 5’-AATTATCAGACTGCTGAGGCCATCACAAAGG-3’

(5) L_1': 5’-GATGGCCTCAGCAGT-3’

(6) A_2: 5’-GCTTATCAGACTAAAAA-NH_2-3’

(7) B_2: 5’-CCATCACAAACCAAGGGTAGGGCGGGTTGGGAAAAA-3’

(8) T_2: 5’-AGTCTGATAAGCAGGTTTGTGATGG-3’

(9) T_1-cali: 5’-GGTTTGTGATGGAAGTCTGATAAGC-3’

Characterizations

Ultraviolet-visible (UV-vis) spectrum was recorded from UV-2450 spectrophotometer (Shimadzu) to calibrate the concentration of DNA strands. Fluorescence signal and

Chemiluminescence spectra was collected on a Cary Eclipse Fluorometer (Varian Inc.). Scanning electron microscopy (SEM) images was acquired on Extra-High-Resolution Scanning Electron Microscope Magellan 400L (ThermoFisher). X-Ray diffraction (XRD) tests was conducted on X-Ray Powder Diffractometer D8 Advance (Bruker AXS). Confocal fluorescence microscopy images were obtained on the Olympus FV3000 confocal laser-scanning microscope.

Preparation of glucose oxidase (GOx)-loaded ZIF-90 NMOFs

The preparation of GOx-loaded ZIF-90 NMOFs followed the enzyme-friendly water-based method according to previous reports with slight modifications. Firstly, 2-ICA (38.4 mg, 0.4 mmol) and PVP (40 mg) was dissolved in a mixture of ethanol/water (ethanol 0.2 mL and water 1.8 mL) by sonicating and heating. Afterwards, the resulting clear yellow solution was cooled down for 4 minutes, and then 75μL of 100 μM GOx (1.2 mg, 190 units-190 U) was added to the cooled mixture of 2-ICA and PVP. Subsequently, 1 mL of $Zn(NO_3)_2{\bullet}6H_2O$ (29.95 mg, 0.1 mmol) in water was added to the mixture of 2-ICA, PVP, and GOx under magnetic stirring. The clear yellow solution turned turbid in 1 minute. After stirring for 1 hour, the GOx-loaded ZIF-90 NMOFs was centrifuged (4000 rpm, 5 minutes) and washed by water for 3 times to get purified NMOFs.

For the preparation of unloaded ZIF-90, the procedures were the same except that GOx was not added.

For the preparation of different amount of GOx-loaded ZIF-90, the procedure were the same except that GOx was added by different amount (95 U, or 380 U).

Functionalize of GOx/ZIF-90 NMOFs with amino modified DNA strands (A_1 orA_2)

1 mL of 1 mg/mL GOx-loaded ZIF-90 NMOFs in water was mixed with 50 μL of 400 μM A_1 or A_2, and then vortexed for 24 hours to allow efficient conjugation of the amino group of DNA strands and aldehyde group of the ZIF-90 NMOFs. After that, the mixture was centrifuged (10000 rpm/5 minutes) and washed by water for 3 times to remove unreacted DNA strands.

Preparation of FITC-labeled GOx

100 μL of 100 μM GOx (in water) was mixed with 50 μL of 1 mM FITC (in DMSO), in a 10 mM of phosphate buffer (pH 9, with final volume of 500 μL). The mixture was vortexed, and kept at 4 °C for overnight. After that, the mixture was purified using an Amicon centrifugal filter unit (10 kDa) (8000 g/10 min) for 3 times to remove unreacted FITC and get FITC-labeled GOx.

Preparation and measurement of the dissipative bioreactor.

For the Nt.BbvCI nickase dictated dissipative system, the concentration of A_1 (**1**) (modified on the GOx-loaded ZIF-90 NMOFs), B_1 (**2**), T_1 (**3**), L_1 (**4**) remained unchanged, and the concentration of fuel L_1' (**5**) and enzyme Nt.BbvCI was tuned to probe experimental effects and validate the simulated temporal dynamic concentration changes of supramolecular bioreactor based on the theoretical model and simulated kinetic parameters. In a typical experiment shown in Figure 3A, 100 μg/mL A_1 (**1**)-modified GOx-loaded ZIF-90 NMOFs, 1 μM B_1 (**2**), 2 μM T_1 (**3**), 2 μM L_1 (**4**), and 200 nM hemin was mixed in rCutsmart buffer (1x) and incubated at 25 °C for 2 h to equilibrate. After that, 0.046 μM enzyme Nt.BbvCI and 3 μM fuel L_1' (**5**) was added to

the mixture. The transient, dynamic formation of (**1**)+(**2**)/(**3**) supramolecular structure then proceeded at 25 °C. At different time-intervals (t = 0/1/2/4/6/8/10 h), the superfluous mixture (800 μL) of dynamic bioreactor was taken out 100 μL, and added with 20 mM glucose and 50 μM Amplex-Red or 100 μM luminol for recording the signal of fluorescent Resorufin or chemiluminescence of luminol.

For Exo III dictated dissipative system, the concentrations of A_2 (**6**) (modified on the GOx-loaded ZIF-90 NMOFs), B_2 (**7**) remained unchanged, and the concentrations of fuel T_2 (**8**) and enzyme Exo III was tuned to probe experimental effects and validate the simulated temporal dynamic concentration changes of supramolecular bioreactor based on the theoretical model and simulated kinetic parameters. In a typical experiment shown in Figure 5A, 100 μg/mL A_2 (**6**)-modified GOx-loaded ZIF-90 NMOFs, 1 μM B_2 (**7**) and 200 nM hemin was mixed in rCutsmart buffer (1x) and incubated at 25 °C for 2 h to equilibrate. After that, 0.4 μM enzyme Exo III and 3 μM fuel T_2 (**8**) was added to the mixture. The transient, dynamic formation of (**6**)+(**7**)/(**8**) supramolecular structure then proceeded at 25 °C. At different time-intervals (t = 0/1/2/4/6/8/10 h), the superfluous mixture (800 μL) of dynamic bioreactor was taken out 100 μL, and added with 20 mM glucose and 50 μM Amplex-Red or 100 μM luminol for recording the signal of fluorescent Resorufin or chemiluminescence of luminol.

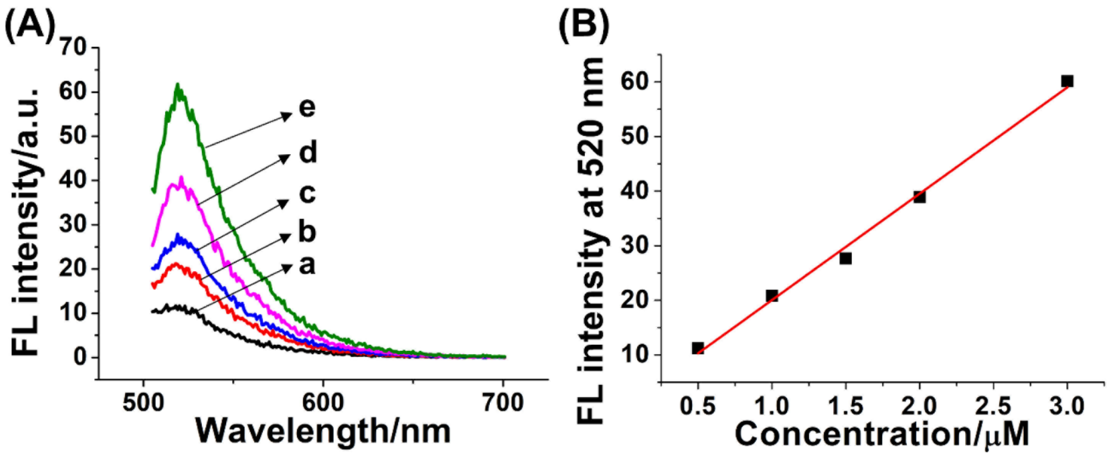

Figure S1. (A) Fluorescence spectra of FITC-labeled GOx at different concentrations: a, 0.5 μM; b, 1 μM; c, 1.5 μM; d, 2 μM; e, 3 μM. (Excitation: 495 nm) (B) Linear calibration curve (FL intensity-concentration) of fluorescent FITC-labeled GOx derived from Figure S1(A).

Determination of the loading amount of enzyme by FITC-labeled GOx-loaded ZIF-90 NMOFs.

The FITC-labeled GOx have similar size with GOx, and is supposed to be similarly loaded into ZIF-90 NMOFs. Here we used same amount of FITC-labeled GOx to prepare enzyme-loaded ZIF-90 NMOFs. After formation of NMOFs, centrifugation was conducted to purify FITC-labeled GOx-loaded ZIF-90 NMOFs. Unloaded FITC-labeled GOx was remained in the supernatant during centrifugation and washing. The amount of unloaded FITC-labeled GOx was determined by a proper fluorescent calibration curve, Figure S1 A and B. Knowing the input and unloaded amount of FITC-labeled GOx, the loading amount of FITC-labeled GOx in ZIF-90 NMOFs is estimated to be 82 μg/mg NMOFs.

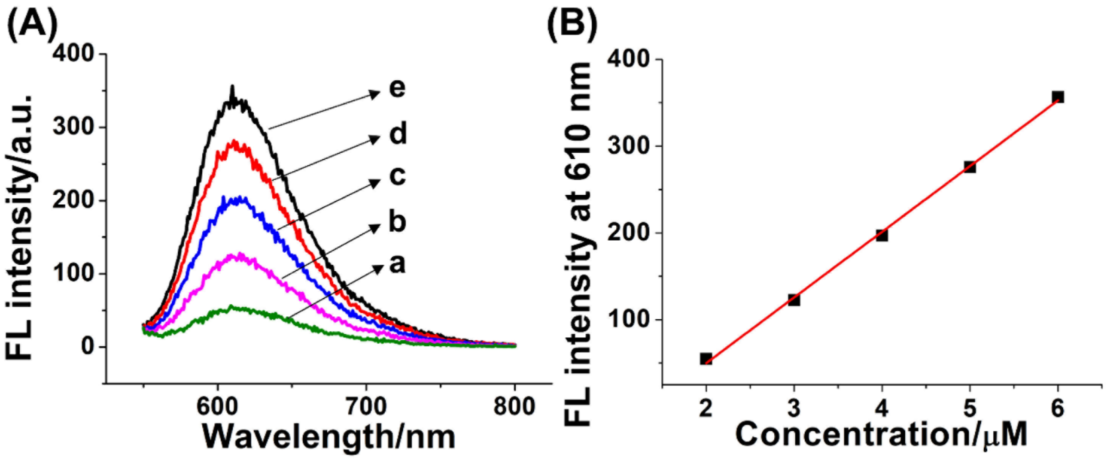

Figure S2. (A) Fluorescence spectrum of GelRed DNA staining dye (10x) in the presence of different concentration of DNA strand A_1: a, 2 μM; b, 3 μM; c, 4 μM; d, 5 μM; e, 6 μM. (Excitation: 530 nm) (B) Linear calibration curve (FL intensity-concentration) of DNA strand A_1 stained by GelRed derived from Figure S2(A).

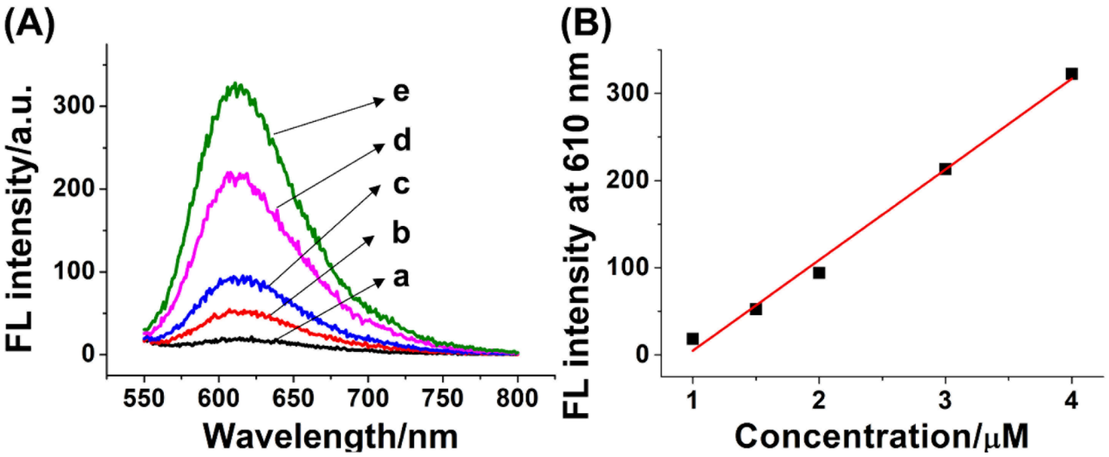

Figure S3. (A) Fluorescence spectrum of GelRed DNA staining dye (10x) in the presence of different concentration of DNA strand A_2: a, 1 μM; b, 1.5 μM; c, 2 μM; d, 3 μM; e, 4 μM. (Excitation: 530 nm) (B) Linear calibration curve (FL intensity-concentration) of DNA strand A_2 stained by GelRed derived from Figure S3(A).

Determination of the surface coverage of DNA strands (A_1 or A_2)

After the purification of the reacted DNA strands with GOx-loaded ZIF-90 NMOFs by centrifugation, the concentration of DNA strands in the removed

supernatant was determined by a calibration curve of fluorescent signal by the binding of DNA strands A_1 and A_2 with a GelRed DNA staining dye, Figure S2 and S3, respectively. Knowing the amount of DNA strands that we input and washed away, the surface coverage of DNA strands (A_1 or A_2) could be estimated to be about 10 nmol/mg NMOFs.

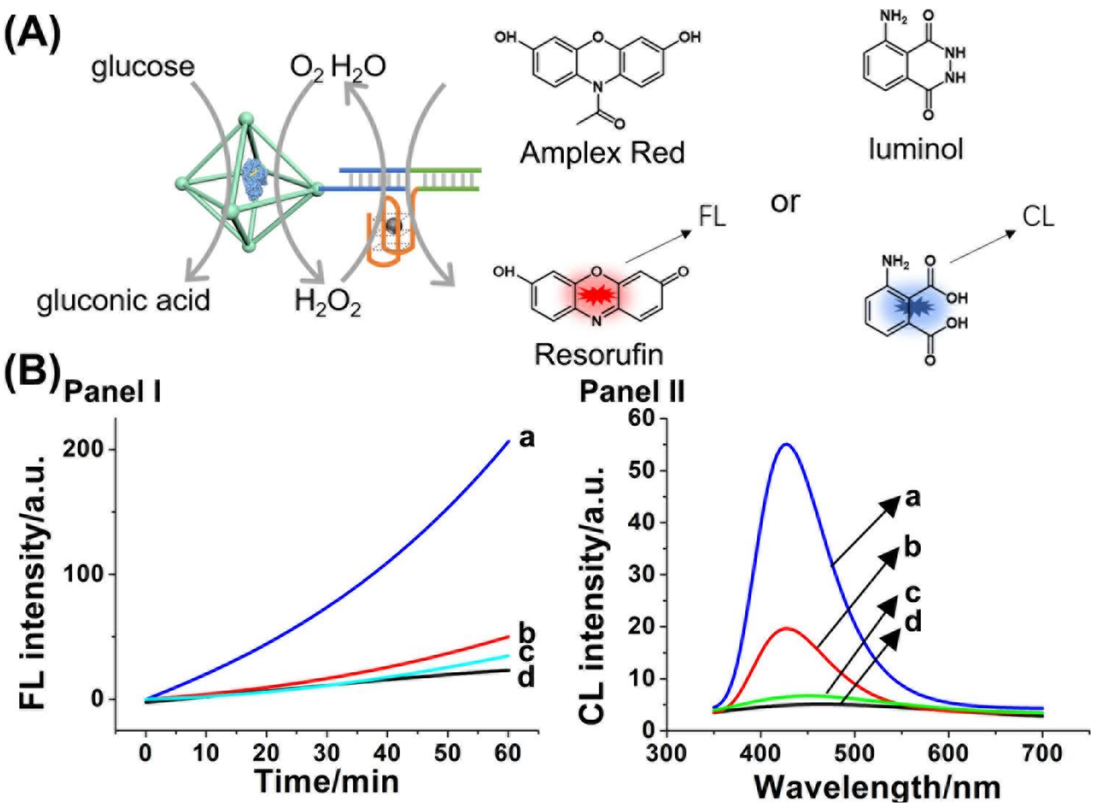

Figure S4. (A) Schematic presentation of biocatalytic cascades performed by the (**1**)+(**2**)/(**3**) supramolecular GOx-loaded ZIF-90 NMOFs/hemin-G-quadruplex bioreactor. (B) Biocatalytic cascades driving the oxidation of Amplex-Red to fluorescent Resorufin (Panel I) or generation of chemiluminescence of luminol (Panel II) by (curve a) (**1**)+(**2**)/(**3**) supramolecular GOx-loaded ZIF-90 NMOFs/hemin-G-quadruplex bioreactor, (curve b) separated GOx-loaded ZIF-90 NMOFs constituent and hemin-G-quadruplex constituent, (curve c) (**1**)+(**2**)/(**3**) supramolecular bioreactor withour K^+, and (curve d) (**1**)+(**2**)/(**3**) supramolecular bioreactor control experiment without glucose.

The biocatalytic cascades performed by the (1)+(2)/(3) supramolecular bioreactor assembled in Figure 1A

The successful construction of (**1**)+(**2**)/(**3**) supramolecular GOx-loaded ZIF-90

NMOFs/hemin-G-quadruplex bioreactor is characterized by the efficient biocatalytic cascades of glucose-driven H_2O_2-channeled oxidation of Amplex-Red or generation of chemiluminescence. As shown in Figure S4A, the glucose is aerobically oxidized by GOx-loaded ZIF-90 NMOFs to form gluconic acid and H_2O_2, and the H_2O_2 is spatial-proximately channeled to the hemin-G-quadruplex assembled with the A_1 (**1**) modified on the NMOFs and used to oxidize Amplex-Red to fluorescent Resorufin or generate chemiluminescence by oxidation of luminol. That is, the spatial confinement of GOx and hemin-G-quadruplex catalytic constituents leads to the effective biocatalytic cascades.[1] Figure S4B Panel I demonstrated enhanced efficient spatial-proximately biocatalytic cascade of (**1**)+(**2**)/(**3**) supramolecular structure for the oxidation of Amplex-Red to fluorescent Resorufin (curve a) compared to the separated constituents (curve b), control system without K^+ (curve c) and control system without glucose (curve d). Figure S4B Panel II demonstrated enhanced efficient spatial-proximately biocatalytic cascade of (**1**)+(**2**)/(**3**) supramolecular structure for the oxidation of luminol to generate chemiluminescence (curve a) compared to the separated constituents (curve b), control system without K^+ (curve c) and control system without glucose (curve d).

Effect of GOx loading amount on the transient GOx-loaded ZIF-90/hemin-G-quadruplex biocatalytic cascade

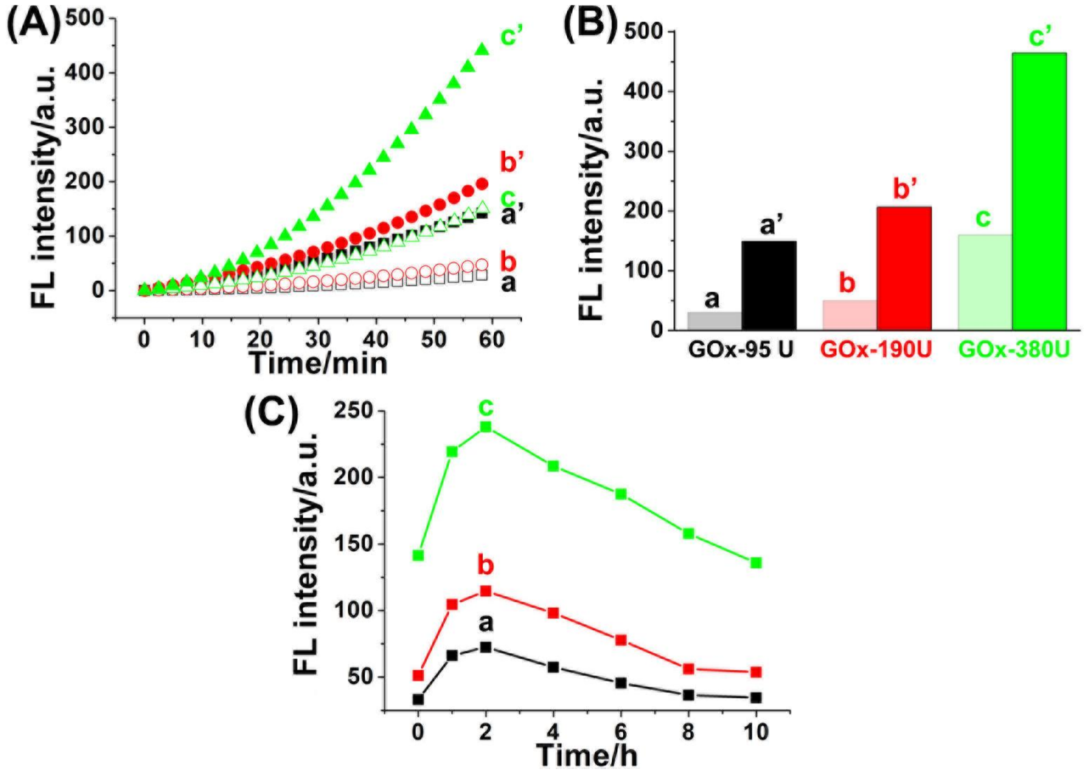

Figure S5. (A) Time-dependent fluorescence changes of Resorufin generated by (**1**)+(**2**)/(**3**) supramolecular GOx-loaded ZIF-90 NMOFs/hemin-G-quadruplex bioreactor at different state with different loading amount of GOx: GOx-95 U (curve a-separated state, curve a'-assembled state), GOx-190 U (curve b-separated state, curve b'-assembled state), and GOx-380 U (curve c-separated state, curve c'-assembled state). (B) Fluorescence intensity of Resorufin of (**1**)+(**2**)/(**3**) supramolecular GOx-loaded ZIF-90 NMOFs/hemin-G-quadruplex bioreactor at different state with different loading amount of GOx derived from Figure S5(B). (C) Temporal, transient, fluorescence intensity changes of the (**1**)+(**2**)/(**3**) supramolecular bioreactor with different loading amount of GOx: GOx-95 U (curve a), GOx-190 U (curve b), and GOx-380 U (curve c), upon operation of the dynamic reaction module (Figure 2, Panel I) at conditions: A_1-

(**1**), 1 μM; B_1-(**2**), 1 μM; T_1/L_1-(**3**)/(**4**), 2 μM; L_1'-(**5**), 3 μM; Nt.BbvCI, 0.046 μM.

The efficacies of the transient, temporal GOx-loaded ZIF-90/hemin-G-quadruplex bioreactor are controlled by the loading degrees of the frameworks with GOx. Figure S5(A) and S5(B) depicts the switchable time-dependent fluorescence intensities of the Resorufin generated by the NMOFs framework shown in Figure 2, Panel I, using different loading degrees of GOx, where (a), (b), and (c) show the fluorescence intensities generated by the separated GOx-loaded NMOFs (GOx-95 U, GOx-190 U, and GOx-380 U) and the hemin/G-quadruplex constituents, and (a'), (b'), and (c') depict the fluorescence intensities by the analog conjugated GOx-loaded ZIF-90/hemin-G-quadruplex frameworks. As the loading of GOx is higher, the biocatalytic cascades are enhanced, yet the background signal of the separated constituents is intensified. Figure S5(C) depict the transient operation of the differently loaded GOx/hemin-G-quadruplex ZIF-90 frameworks. As the loading of GOx in the framework is higher, the concentrations of the transiently formed Resorufin product, generated by the transient intermediate (**1**)+(**2**)/(**3**) supramolecular GOx-loaded ZIF-90 NMOFs/hemin-G-quadruplex bioreactor, are higher. Yet the depletion rates stimulated by the fuel-strands and nickase are unaffected.

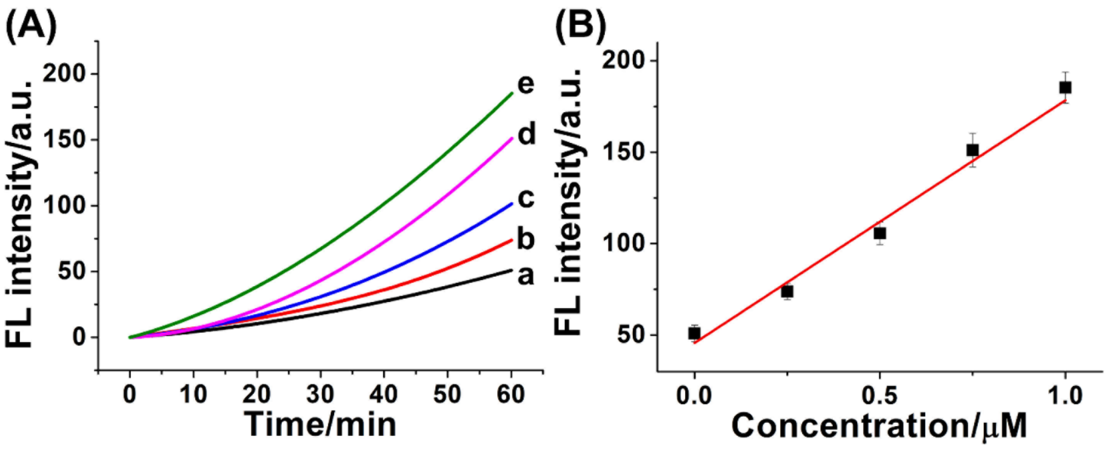

Figure S6. (A) Biocatalytic cascade oxidation of Amplex-Red to fluorescent Resorufin by (**1**)+(**2**) constituents (100 μg/mL (**1**)-modified GOx-loaded ZIF-90 NMOFs, corresponding to 1μM of (**1**), and 1μM of (**2**)) in the presence of variable concentrations of T_1-cali: a, 0 μM; b, 0.25 μM; c, 0.5 μM; d, 0.75 μM; e, 1 μM. The T_1-cali could bind (**1**)+(**2**) constituents together with almost 100% efficiency according the Nupack analysis result. (B) Calibration curve relating the fluorescent Resorufin to the concentration of (**1**)+(**2**)/(**3**) supramolecular bioreactor derived from Figure S6(A).

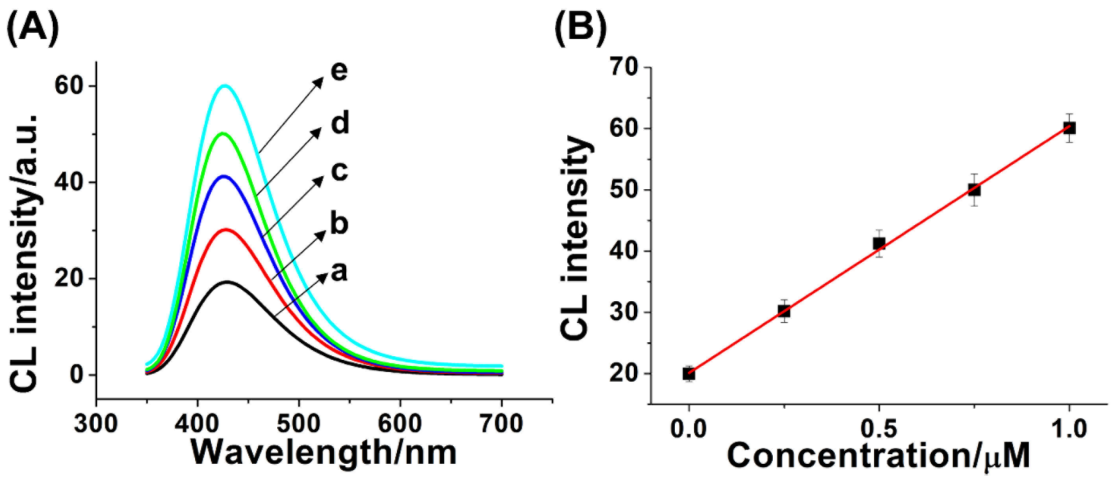

Figure S7. (A) Biocatalytic cascade oxidation of luminol to generation of chemiluminescence by (**1**)+(**2**) constituents (100 μg/mL (**1**)-modified GOx-loaded ZIF-90 NMOFs, corresponding to 1 μM of (**1**), and 1 μM of (**2**)) in the presence of variable concentrations of T_1-cali: a, 0 μM; b, 0.25 μM; c, 0.5 μM; d, 0.75 μM; e, 1 μM. The T_1-cali could bind (**1**)+(**2**) constituents together with almost 100% efficiency according the Nupack analysis result. (B) Calibration curve relating the chemiluminescence intensity to the concentration of (**1**)+(**2**)/(**3**) supramolecular bioreactor derived from Figure S7(A).

Kinetic equations of the dissipative bioreactor system shown in Figure 2:

(1) $L_1 + T_1 \underset{K_{-1}}{\overset{K_1}{\rightleftarrows}} L_1T_1$

(2) $L_1T_1 + L'_1 \underset{K_{-2}}{\overset{K_2}{\rightleftarrows}} L_1L'_1 + T_1$

(3) $T_1 + A_1 + B_1 \underset{K_{-3}}{\overset{K_3}{\rightleftarrows}} T_1A_1B_1$

(4) $L_1L'_1 + E \underset{K_{-4}}{\overset{K_4}{\rightleftarrows}} L_1L'_1E$

(5) $L_1L'_1E \overset{K_5}{\rightarrow} E + L_1L'_{1-1}L'_{1-2}$

(6) $L_1L'_{1-1}L'_{1-2} \underset{K_{-6}}{\overset{K_6}{\rightleftarrows}} L_1 + L'_{1-1} + L'_{1-2}$

Derivatives:

(7) $\frac{dL_1}{dt} = K_{-1}[L_1T_1] - K_1[T_1][L_1] + K_6[L_1L'_{1-1}L'_{1-2}] - K_{-6}[L_1][L'_{1-1}][L'_{1-2}]$

(8) $\frac{dT_1}{dt} = K_{-1}[L_1T_1] - K_1[T_1][L_1] + K_2[L_1T_1][L'_1] - K_{-2}[L_1L'_1][T_1] +$

$K_{-3}[T_1A_1B_1] - K_3[T_1][A_1][B_1]$

(9) $\frac{dL_1T_1}{dt} = K_1[T_1][L_1] - K_{-1}[L_1T_1] + K_{-2}[L_1L'_1][T_1] - K_2[L_1T_1][L'_1]$

(10) $\frac{dL'_1}{dt} = K_{-2}[L_1L'_1][T_1] - K_2[L_1T_1][L'_1]$

(11) $\frac{dL_1L'_1}{dt} = K_2[L_1T_1][L'_1] - K_{-2}[L_1L'_1][T_1] + K_{-4}[L_1L'_1E] - K_4[L_1L'_1][E]$

(12) $\frac{dA_1}{dt} = K_{-3}[T_1A_1B_1] - K_3[T_1][A_1][B_1]$

(13) $\frac{dB_1}{dt} = K_{-3}[T_1A_1B_1] - K_3[T_1][A_1][B_1]$

(14) $\frac{dT_1A_1B_1}{dt} = K_3[T_1][A_1][B_1] - K_{-3}[T_1A_1B_1]$

(15) $\frac{dE}{dt} = K_{-4}[L_1L'_1E] - K_4[L_1L'_1][E] + K_5[L_1L'_1E]$

(16) $\frac{dL_1L'_1E}{dt} = K_4[L_1L'_1][E] - K_{-4}[L_1L'_1E] - K_5[L_1L'_1E]$

(17) $\frac{dL_1L'_{1-1}L'_{1-2}}{dt} = K_5[L_1L'_1E] + K_{-6}[L_1][L'_{1-1}][L'_{1-2}] - K_6[L_1L'_{1-1}L'_{1-2}]$

(18) $\frac{dL'_{1-1}}{dt} = K_6[L_1L'_{1-1}L'_{1-2}] - K_{-6}[L_1][L'_{1-1}][L'_{1-2}]$

(19) $\frac{dL'_{1-2}}{dt} = K_6[L_1L'_{1-1}L'_{1-2}] - K_{-6}[L_1][L'_{1-1}][L'_{1-2}]$

Figure S8. Computational simulation of the dynamic, transient, dissipative bioreactor

system shown in Figure 2. The kinetic scheme of the reactions associated with the time-dependent concentration changes of the (**1**)+(**2**)/(**3**) supramolecular structure is summarized in above equations. From the experimental derived concentration changes of the (**1**)+(**2**)/(**3**) supramolecular structure, we computationally simulated the kinetics parameters of time-dependent concentration changes by using Matlab R2019b.

Computational details about the simulation using Matlab R2019b

The rate equation corresponding to the respective sub-reactions were defied as equations (7-19). These rate equations were coded in the Matlab R2019b software while providing K_i/K_{-i} values of the set of rate equations, together with the initial concentrations of the constituents in the system at t = 0 min (the K_i/K_{-i} values were based on literature values of related duplexes and provide approximate inputs), resulting in the first dynamic curves corresponding to the temporal concentrations of the constituents. As the experimental temporal concentrations of the constituents are read as a data matrix, the nonlinear least-squares solver (Lsqcurvefit) embedded in Matlab software compares the first round of simulated data experimental data and initiates an optimization iteration under the optimization limits: 'StepTolerance',1e-90, 'FunctionTolerance',1e-15, 'OptimalityTolerance', 1e-20, 'MaxFunctionEvaluation', 10000). This simulation procedure is processed till a satisfactory fit between the simulated results and experimental results is obtained. Usually, the maximum number of 500 stimulated iterations yield a satisfactory fit between the computationally-simulated results and experimental data leading to a set of rate-constants that are supposed to follow the kinetic model. To support the computationally simulated results,

it is well desirable to compare one (or more) experimentally validated rate constant to the simulated values. Alternatively, to avoid a possible meaningless set of computationally derived rate constants, the significance of the computational results may be supported by predicting the temporal concentrations of the constituents at different auxiliary conditions and validation of the predicted values by experiments.

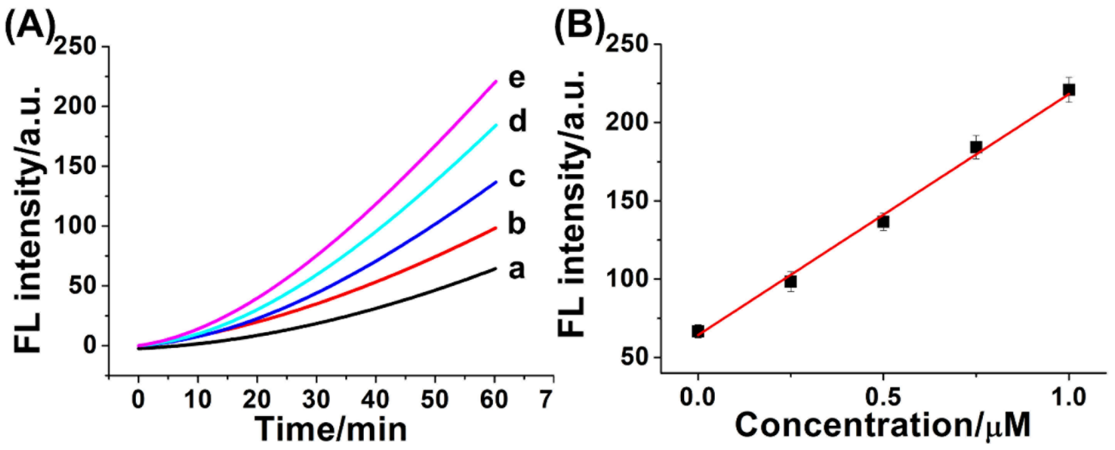

Figure S9. (A) Biocatalytic cascade oxidation of Amplex-Red to fluorescent Resorufin by (**6**)+(**7**) constituents (100 μg/mL (**6**)-modified GOx-loaded ZIF-90 NMOFs, corresponding to 1μM of (**6**), and 1μM of (**7**)) in the presence of variable concentrations of (**8**): a, 0 μM; b, 0.25 μM; c, 0.5 μM; d, 0.75 μM; e, 1 μM. (B) Calibration curve relating the fluorescent Resorufin to the concentration of (**6**)+(**7**)/(**8**) supramolecular bioreactor derived from Figure S9(A).

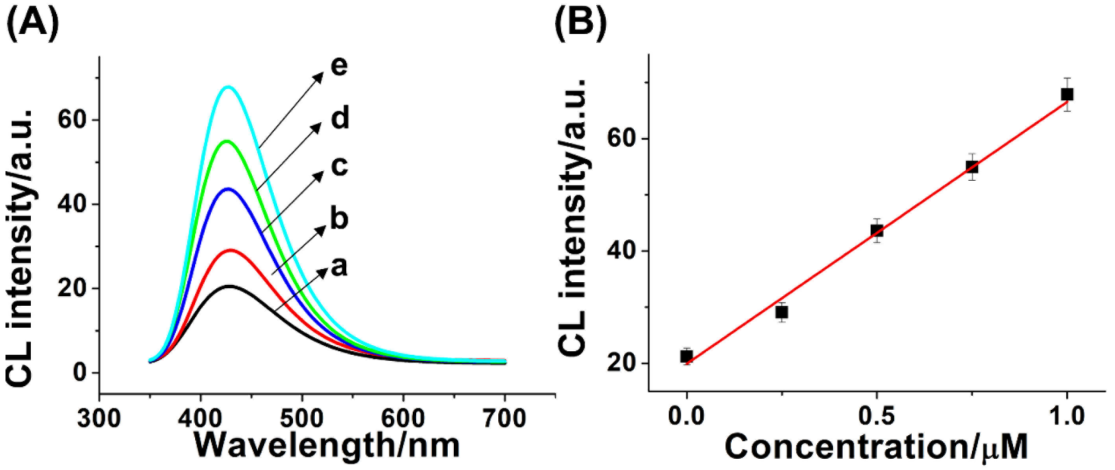

Figure S10. (A) Biocatalytic cascade oxidation of luminol to generate chemiluminescence by (**6**)+(**7**) constituents (100 μg/mL (**6**)-modified GOx-loaded ZIF-90 NMOFs, corresponding to 1μM of (**6**), and 1μM of (**7**)) in the presence of variable concentrations of (**8**): a, 0 μM; b, 0.25 μM; c, 0.5 μM; d, 0.75 μM; e, 1 μM. (B) Calibration curve relating the intensity of chemiluminescence to the concentration of (**6**)+(**7**)/(**8**) supramolecular bioreactor derived from Figure S10(A).

Kinetic equations of the dissipative bioreactor system shown in Figure 4:

(1) $T_2 + A_2 + B_2 \underset{K_{-1}}{\overset{K_1}{\rightleftarrows}} T_2A_2B_2$

(2) $T_2A_2B_2 + E \underset{K_{-2}}{\overset{K_2}{\rightleftarrows}} ET_2A_2B_2$

(3) $ET_2A_2B_2 \overset{K_3}{\rightarrow} E + T_2'A_2B_2$

(4) $T_2'A_2B_2 \underset{K_{-4}}{\overset{K_4}{\rightleftarrows}} T_2' + A_2 + B_2$

Derivatives:

(5) $\frac{dA_2}{dt} = K_{-1}[T_2A_2B_2] - K_1[T_2][A_2][B_2] + K_4[T_2'A_2B_2] - K_{-4}[T_2'][A_2][B_2]$

(6) $\frac{dB_2}{dt} = K_{-1}[T_2A_2B_2] - K_1[T_2][A_2][B_2] + K_4[T_2'A_2B_2] - K_{-4}[T_2'][A_2][B_2]$

(7) $\frac{dT_2}{dt} = K_{-1}[T_2A_2B_2] - K_1[T_2][A_2][B_2]$

(8) $\frac{dT_2A_2B_2}{dt} = K_1[T_2][A_2][B_2] - K_{-1}[T_2A_2B_2] + K_{-2}[T_2A_2B_2E] - K_2[T_2A_2B_2][E]$

(9) $\frac{dE}{dt} = K_{-2}[T_2A_2B_2E] - K_2[T_2A_2B_2][E] + K_3[T_2A_2B_2E]$

(10) $\frac{dT_2A_2B_2E}{dt} = K_2[T_2A_2B_2][E] - K_{-2}[T_2A_2B_2E] - K_3[T_2A_2B_2E]$

(11) $\frac{dT_2'A_2B_2}{dt} = K_3[T_2A_2B_2E] + K_{-4}[T_2'][A_2][B_2] - K_4[T_2'A_2B_2]$

(12) $\frac{dT_2'}{dt} = K_4[T_2'A_2B_2] - K_{-4}[T_2'][A_2][B_2]$

Figure S11. Computational simulation of the dynamic, transient, dissipative bioreactor system shown in Figure 4. The kinetic scheme of the reactions associated with the time-dependent concentration changes of the (**6**)+(**7**)/(**8**) supramolecular structure is summarized in above equations. From the experimental derived concentration changes of the (**6**)+(**7**)/(**8**) supramolecular structure, we computationally simulated the kinetics parameters of time-dependent concentration changes by using Matlab R2019b.

Table S1. Rate constants derived from the computational simulation of the dissipative bioreactor system shown in Figure 2.

K_1	16.9 $\mu M^{-1} \bullet min^{-1}$	K_3	9.1 $\mu M^{-2} \bullet min^{-1}$	K_5	0.5 min^{-1}
K_{-1}	$1*10^{-4}$ min^{-1}	K_{-3}	0.02 min^{-1}	K_6	8.7 min^{-1}
K_2	0.9 $\mu M^{-1} \bullet min^{-1}$	K_4	11.8 $\mu M^{-1} \bullet min^{-1}$	K_{-6}	0.02 $\mu M^{-2} \bullet min^{-1}$
K_{-2}	11.3 $\mu M^{-1} \bullet min^{-1}$	K_{-4}	0.4 min^{-1}		

Table S2. Rate constants derived from the computational simulation of the dissipative bioreactor system shown in Figure 4.

K_1	10.5 $\mu M^{-2} \bullet min^{-1}$	K_3	0.09 min^{-1}
K_{-1}	0.01 min^{-1}	K_4	13.6 min^{-1}
K_2	3.4 $\mu M^{-1} \bullet min^{-1}$	K_{-4}	0.01 $\mu M^{-2} \bullet min^{-1}$
K_{-2}	0.05 min^{-1}		

References:

(1) Vázquez-González, M.; Wang, C.; Willner, I., Biocatalytic cascades operating on macromolecular scaffolds and in confined environments. Nat. Catal. 2020, 3 (3), 256-273.
